# Time Distortion in Parkinsonism

**DOI:** 10.3389/fnins.2021.648814

**Published:** 2021-03-19

**Authors:** Yasuo Terao, Motoyasu Honma, Yuki Asahara, Shin-ichi Tokushige, Toshiaki Furubayashi, Tai Miyazaki, Satomi Inomata-Terada, Ayumi Uchibori, Shinji Miyagawa, Yaeko Ichikawa, Atsuro Chiba, Yoshikazu Ugawa, Masahiko Suzuki

**Affiliations:** ^1^Department of Medical Physiology, School of Medicine, Kyorin University, Tokyo, Japan; ^2^Department of Physiology, School of Medicine, Showa University, Tokyo, Japan; ^3^Department of Neurology, The Jikei University Katsushika Medical Center, Tokyo, Japan; ^4^Department of Neurology, Kyorin University Hospital, Tokyo, Japan; ^5^Graduate School of Health and Environment Science, Tohoku Bunka Gakuen University, Sendai, Japan; ^6^Department of Human Neurophysiology, School of Medicine, Fukushima Medical University, Fukushima, Japan

**Keywords:** time perception, Parkinson’s disease, basal ganglia, dopamine, progressive supranuclear palsy

## Abstract

Although animal studies and studies on Parkinson’s disease (PD) suggest that dopamine deficiency slows the pace of the internal clock, which is corrected by dopaminergic medication, timing deficits in parkinsonism remain to be characterized with diverse findings. Here we studied patients with PD and progressive supranuclear palsy (PSP), 3–4 h after drug intake, and normal age-matched subjects. We contrasted perceptual (temporal bisection, duration comparison) and motor timing tasks (time production/reproduction) in supra- and sub-second time domains, and automatic versus cognitive/short-term memory–related tasks. Subjects were allowed to count during supra-second production and reproduction tasks. In the time production task, linearly correlating the produced time with the instructed time showed that the “subjective sense” of 1 s is slightly longer in PD and shorter in PSP than in normals. This was superposed on a prominent trend of underestimation of longer (supra-second) durations, common to all groups, suggesting that the pace of the internal clock changed from fast to slow as time went by. In the time reproduction task, PD and, more prominently, PSP patients over-reproduced shorter durations and under-reproduced longer durations at extremes of the time range studied, with intermediate durations reproduced veridically, with a shallower slope of linear correlation between the presented and produced time. In the duration comparison task, PD patients overestimated the second presented duration relative to the first with shorter but not longer standard durations. In the bisection task, PD and PSP patients estimated the bisection point (BP50) between the two supra-second but not sub-second standards to be longer than normal subjects. Thus, perceptual timing tasks showed changes in opposite directions to motor timing tasks: underestimating shorter durations and overestimating longer durations. In PD, correlation of the mini-mental state examination score with supra-second BP50 and the slope of linear correlation in the reproduction task suggested involvement of short-term memory in these tasks. Dopamine deficiency didn’t correlate significantly with timing performances, suggesting that the slowed clock hypothesis cannot explain the entire results. Timing performance in PD may be determined by complex interactions among time scales on the motor and sensory sides, and by their distortion in memory.

## Introduction

The widely held scalar expectancy theory (SET) assumes that temporal information processing in the mind consists of different processes, such as the clock (pacemaker), switch, memory, and decision processes (Gibbon, 1977; Gibbon et al., 1984). The duration of time is perceived by the accumulated pulses of the clock that are encoded into working memory and, with time, are transformed into more enduring internal temporal representations that come to be stored in long-term memory. The perception of time is determined by the decision process that compares the count of pulses accumulated with the reference time representation stored in memory.

The motor system is intricately associated with the processing of temporal information in the brain. Among them, the striatum and dopamine have been assigned a central role in processing explicit timing (Jones and Jahanshahi, 2011, 2014a, 2015; Harrington and Rao, 2015). Early animal studies have provided evidence that the main function of the basal ganglia is to determine the presumed pacemaker speed by showing that clock processes depend on the neurotransmission of dopamine. For example, when animals trained by being rewarded for pressing a button at a fixed time after a visual signal is presented were administered dopamine blockers, they tended to respond with a longer response time than before (Maricq and Church, 1983; Meck, 1998; Buhusi and Meck, 2002; Matell and Meck, 2004; Matell et al., 2006; Matell, 2014).

Clinically, patients with Parkinson’s disease (PD), a basal ganglia disorder involving dopamine deficiency, have provided insights into their influence on temporal processing (Koch et al., 2009; Jones and Jahanshahi, 2011, 2014a, 2015; Magalhães et al., 2018). Since PD patients in general present with bradykinesia, that is, slowness of movement, it was speculated that slowness may also involve the temporal processing of the mind. If we postulate that the mind uses an “internal” clock that ticks at a regular rate to perceive the passage of time, it would tick slower in the presence of dopamine deficiency. Temporal processing deficits have been studied using timing tasks, including production and reproduction, time estimation, and time discrimination in both normal subjects and patients with neurological disorders. In line with the results of animal studies, early studies showed that PD patients underestimate the duration of given stimuli (e.g., 1–60 s) (Pastor et al., 1992a; Lange et al., 1995) while they over-reproduce the durations (Lange et al., 1995; Smith et al., 2007; Koch et al., 2008; Wild-Wall et al., 2008), a pattern expected from the slowed clock hypothesis. Furthermore, this was shown to be normalized by the administration of l-dopa given as a treatment (Pastor et al., 1992a,b; O’Boyle et al., 1996). Together, the findings in both humans and animals indicate that dopamine depletion slows the pace of the internal clock, which can be corrected by administering dopaminergic drugs.

Despite its intuitive appeal, however, later studies have not necessarily replicated evidence in support of the slowed clock hypothesis in the presence of dopamine deficiency. Although no or little impairment in the time estimation and time production tasks is described, normal performance has been found in several different tests of time perception (Riesen and Schnider, 2001; Perbal et al., 2005; Wearden et al., 2008; Jones and Jahanshahi, 2015).

Although the above studies are based on the assumption that the “internal clock” ticks at a fixed constant pace, some studies report observations that can be accounted for only by postulating a change in the pace of a “regularly ticking” clock. Repetitively timed movements in PD can be overproduced, especially for sub-second intervals (Pastor et al., 1992a; Harrington et al., 1998a; Elsinger et al., 2003; Jones and Jahanshahi, 2009; Jones et al., 2011). In contrast, Honma et al. (2016, 2017) showed that when PD patients were required to produce the time indicated by number of seconds (e.g., 1 s, 3 s, ….) by mental counting (i.e., by silently counting at their subjective 1 s intervals to themselves), they overproduced time below 2–3 s and underproduced it when the indicated time was over 4 s. Underproduction of longer time intervals would suggest faster rather than slower ticking of the internal clock whereas overproduction of shorter durations indicates slower rather than faster ticking. Thus, there must be a distortion of time, with under- and overproduction depending on the time span to be processed.

Similar over- and underproduction has been described in a time reproduction task in which subjects were asked to reproduce time durations presented before. Malapani et al. (1998) and Malapani and Ratikin (2003) reported that when PD patients were asked to reproduce time intervals of 8 or 21 s from memory within a task, they overproduced the 8 s interval and underproduced the 21 s interval. Similarly, a within-task overestimation of relatively short intervals and underestimation of relatively long intervals has been confirmed in PD (Koch et al., 2004, 2005, 2008; Jones and Jahanshahi, 2014a). The migration effect in PD cannot be explained in terms of decreased internal timekeeper speed. Instead, since the time span should be stored in the memory even temporarily to perform the task, this distortion (i.e., the overproduction of shorter intervals and underestimation of longer intervals) was considered to be related to memory. More specifically, it was related to the encoding or retrieval of temporal information into and out of short-term memory, which, in fact, correlated with short-term memory scores (i.e., mutual interaction and averaging among memory traces of time intervals during their retrieval) (Malapani et al., 2002; Dušek et al., 2012). This pattern of time distortion is similar to that reported by Honma et al. (2016), but because they compared time production and reproduction tasks to separate time estimation from working memory ability, they concluded that time distortion is guided by the cumulative output of fast cycle counting, independent of short-term memory.

In the present study, using various timing tasks, we investigated the overall pattern of time distortion in PD. Studies have shown a significant correlation between perceptual and motor timing tasks, leading to the assumption of a common neural substrate (Keele et al., 1985; Ivry and Schlerf, 2008; Merchant et al., 2008a,b,c; Bangert et al., 2011). In contrast, since motor responses are intrinsically tied to temporal decisions in various timing tasks, the motor performance of PD patients may also impact the performance of timing tasks, independent of the temporal perception *per se*. To dissociate dysfunctional time perception from the motor components of temporal processing, we mainly used perceptual timing tasks (duration comparison and bisection tasks) that dissociate motor responses from temporal decisions, as well as time production/reproduction tasks, which involve explicit motor components. We refer to these tasks as “perceptual” and “motor” timing tasks in the following.

Since the discrepant findings reported on timing tasks may arise from the use of different time durations, we also implemented timing tasks in both the sub- and supra-second ranges, since timing in the supra-second range is considered to be more cognitive and/or memory-based whereas timing tasks in the sub-second range are considered to be more automatic, that is, either perceptual (i.e., more sensory or iconic) (Perbal-Hatif, 2012) or more directly associated with the motor system (Ivry and Spencer, 2004; Lewis and Miall, 2009). Furthermore, processing time durations in the sub- and supra-second range are suggested to be subserved by distinct neural substrates. Sub-second durations are mainly processed by the frontal operculum, cerebellar hemisphere, and middle and superior temporal gyri, whereas durations in the supra-second range require more cognitive processing by the frontal cortex and the basal ganglia (Gibbon et al., 1997; Lewis and Miall, 2002, 2003a,b, 2009; Ivry and Spencer, 2004; Lewis et al., 2004). Here, the boundary between sub- and supra-second intervals need not necessarily be above and below 1 s. It has been suggested that there is a limit to the span of time that one can integrate and perceive as a ‘perceptual unit’ at around 2–3 s, which has been supported by a number of studies on the temporal reproduction of stimuli (Pöppel, 1978). The boundary may thus lie between 2 and 3 s. Consistently, whereas stimuli are reproduced relatively accurately up to 3 s in temporal reproduction tasks, longer stimuli are incorrectly reproduced (Mates et al., 1994; Claassen et al., 2013; Matsuda et al., 2015; Tolleson C. et al., 2015; Tokushige et al., 2018).

Finally, the time span perceived directly from the stimuli or those that are recalled from stored memory may differ. Since short-term and working memory would be used in a time reproduction task (Perbal-Hatif, 2012), distortions may also arise during memory encoding and retrieval. Thus, we also compared the time production and reproduction tasks to separate time estimation from working memory ability.

Progressive supranuclear palsy is almost indistinguishable from PD in the earliest stage, exhibiting a similar degree of bradykinesia (Litvan et al., 1996; Williams et al., 2005; Phokaewvarangkul and Bhidayasiri, 2019). Since parkinsonism is associated with bradykinesia of movement, we also studied PSP patients in a similar Parkinson’s stage to determine whether their timing task performances differ from PD at similar stages of parkinsonism. Prefrontal dysfunction related to short-term memory will be more affected in PSP. The performance of timing tasks was correlated with the Unified Parkinson’s Disease Rating Scale (UPDRS) motor score and the specific binding rate (SBR) of the striatum in the dopamine transporter single photon emission computed tomography (DAT-SPECT), as an indicator of the dopamine-deficient state. The covariates considered were the age of subjects, disease duration, and the Mini-Mental State Examination (MMSE) score.

## Materials and Methods

### Subjects

Initially, 36 PD patients (age: 71.3 ± 7.6) and 14 PSP patients (age: 73.5 ± 4.9) from the neurological outpatient clinic of Kyorin University and the Jikei University Katsushika Medical Center participated in the main part of the experiment (temporal bisection task, time production task, time reproduction task). Out of these patients, one PD patient and three PSP patients were excluded because they could not follow the instructions of the task. As a result, 35 PD patients (age: 71.3 ± 7.6) and 11 PSP patients (age: 73.5 ± 4.9) in the main part of the experiment. Data from 20 age-matched normal subjects without neurological or other medical disorders (age: 73.0 ± 6.8) were also collected as a normal control (NC) group, with no significant difference in age between PD and PSP patients. In the time production task with a wider range of instructed duration (see below), 14 age-matched healthy volunteers (age: 73.4 ± 4.7) along with 12 PD patients (age: 73.0 ± 4.4, UPDRS-III: 16.9 ± 13.4) were enrolled. In the duration comparison task, 12 PD patients (age: 73.9 ± 5.9, UPDRS-III score 16.4 ± 16.0) along with 15 normal age-matched control subjects (age: 73.2 ± 4.9) participated. The participants in the time production task and the duration comparison task were separate from the participants in the main part of experiment. The subject characteristics are summarized in Table 1.

**TABLE 1:** Subject characteristics.

Task	Normal subjects		PD patients			PSP patients		
	Subjects(M/F)	Age	Subjects(M/F)	Age	UPDRS	Subjects(M/F)	Age	UPDRS
Duration comparison task	15 (6M, 9F)	73.2 ± 4.9	12 (7M, 5F)	73.9 ± 5.9	16.4 ± 16.0	–	–	–
Time bisection task	20 (10M, 10F)	73.0 ± 6.8	35 (14M, 21F)	71.3 ± 7.6	26.0 ± 8.4	11 (8M, 3F)	73.5 ± 4.9	29.3 ± 9.4
Time production task	20 (10M, 10F)	73.0 ± 6.8	35 (14M, 21F)	71.3 ± 7.6	26.0 ± 8.4	11 (8M, 3F)	73.5 ± 4.9	29.3 ± 9.4
Time reproduction task	20 (10M, 10F)	73.0 ± 6.8	35 (14M, 21F)	71.3 ± 7.6	26.0 ± 8.4	11 (8M, 3F)	73.5 ± 4.9	29.3 ± 9.4
Time reproduction task (0.5–10 s)	14 (6M, 8F)	73.4 ± 4.7	12 (4M, 8F)	73.0 ± 4.4	16.9 ± 13.4	–	–	–

All experiments were conducted in accordance with the ethical standards of the Declaration of Helsinki, after the participants provided written informed consent prior to participation. All experimental procedures were approved by the local ethics committee of the Faculty of Medicine, Kyorin University, and the Jikei University Katsushika Medical Center.

The diagnosis of PD and PSP was based on the diagnostic criteria of the Parkinson’s Disease Society Brain Bank and the National Institute of Neurologic Disorders and Stroke–SPSP diagnostic criteria. Clinical follow-up was also consistent with the diagnosis of PD and PSP. When available, the diagnosis was also confirmed by neuroimaging. In 27 PD patients and 9 PSP patients, DAT-SPECT using ^123^I-2β-carbomethoxy-3β(4-iodo-phenyl) tropane (^123^I-β-CIT) and ^123^I-metaiodobenzylguanidine (MIBG) scintigraphy were also performed as part of the clinical assessment. The heart to mediastinum (H/M) ratio in the late phase of MIBG scintigraphy used to confirm the diagnosis of PD (Maruyama et al., 2015) and the specific binding ratio (SBR) of DAT-SPECT averaged across the bilateral basal ganglia (average SBR) were included as clinical covariates in the multiple regression analysis below. The typical clinical features for patient inclusion are summarized in Table 2.

**TABLE 2:** Typical clinical and neuroimaging features for patient inclusion.

Diagnosis	Onset, occurrence	Characteristic clinical findings	MRI	SBR in DAT-SPECT	MIBG* (Late phase H/M ratio)	SPECT*
Parkinson’s disease (PD)	Sporadic occurrence, asymmetric symptoms at onset	Gradual progression, 4–6 Hz rest tremor, rigidity, postural instability, olfactory dysfunction, clear beneficial response to dopaminergic therapy	Normal	Reduced uptake	Reduced	Normal perfusion (mild parieto-occipital hypoperfusion)
Progressive supranuclear palsy (PSP)	Sporadic occurrence, Onset at age 40 or older	Vertical supranuclear palsy), postural instability, akinesia, and cognitive dysfunction	Predominant midbrain atrophy, enlargement of the third ventricle	Reduced uptake	Normal	Hypoperfusion (medial frontal lobe, anterior cingulate, midbrain)

*When available.

The disease severity of PD and PSP patients was assessed by the Hoehn–Yahr scale and the UPDRS motor score. As a gross index of cognitive impairment, the MMSE was performed. Subjects with an MMSE score below 22 and those who could not follow the task instructions were excluded from the analysis.

The PD patients were taking their regular doses of L-dopa alone or in combination with a dopamine agonist (ropinirol or pramipexol). For ethical and clinical reasons, the medication could not be withdrawn completely, but the experiments were conducted at least 3 to 4 h after last drug intake (including L-DOPA) when, based on previous studies, only a minimal change in the performance of saccade tasks (Yugeta et al., 2008; Terao et al., 2011) and timing tasks (Koch et al., 2008) would have persisted. The half-life of dopamine agonists is relatively long and could not be washed out. Also, all PSP patients stopped taking medication (levodopa in all cases) at least 3–4 h before measurements were performed.

### Experimental Procedures

Subjects performed four timing tasks, both motor and perceptual, which were performed according to procedures in previous studies (Honma et al., 2016, 2017, 2018).

### Duration Comparison Task

**FIGURE 1:**
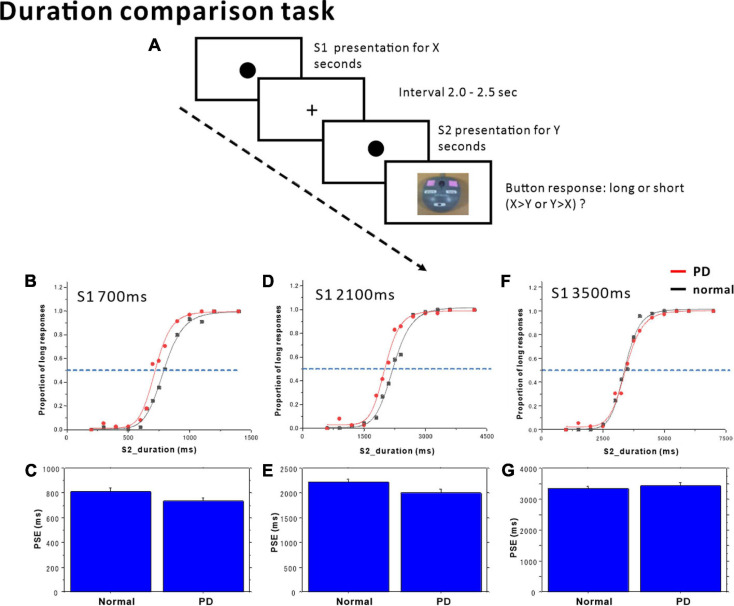
Duration comparison task. **(A)** The subjects compared time durations (S1, S2) presented successively with an interval of 2.0–2.5 s and judged which of the two durations was longer. Subjects responded by pressing one of two buttons, corresponding to long or short responses. The proportion of trials in which subjects judged S2 duration to be longer than S1 duration was plotted as a function of S1 (standard) duration, separately in the **(B)** S1 = 700 ms, **(D)** S1 = 2100 ms, and (**F**) S1 = 3500 ms standard tasks. Red dots: PD patients, Black dots: normal subjects. The scattergrams were fitted to a logistic function. The horizontal dashed lines indicate the 50% level on the ordinate. The cross-point with the curve corresponds to the point of subjective equality (PSE). The PSE was compared between normal subjects and PD patients. **(C)** S1 = 700 ms, **(E)** S1 = 2100 ms, **(G)** S1 = 3500 ms. Error bars give standard errors.

In this task, the subjects (PD patients and normal subjects) compared the duration of tones (S1, S2) presented successively with a randomized interval of 2000–2500 ms between them (Figure 1A). The subjects had to judge whether the duration of S2 was longer or shorter than that of S1 by a button press (long and short). Subjects pressed the “long” button when they judged that S2 was longer than S1, and vice versa. The task was considered to be a perceptual timing task. Accuracy of judgment, but not promptness of response, was emphasized.

The duration of the first standard tone (S1) was fixed within each block of sessions at either 700, 2100, or 3500 ms. The comparison tone S2 had a duration of either 200, 300, 400, 500, 600, 650, 750, 800, 900, 1000, 1100, or 1200 ms in the S1 = 700 ms standard blocks; 600, 900, 1200, 1500, 1800, 1950, 2350, 2400, 2700, 3000, 3300, or 3660 ms in the S1 = 2100 ms standard blocks; and 1000, 1500, 2000, 2500, 3000, 3250, 3750, 4000, 4500, 5000, 5500, 6000, or 7000 ms in the S1 = 3500 ms standard blocks. Each of the S2 durations was presented three times within a single session in a pseudorandomized and counterbalanced order in one block. Implicit or explicit counting was discouraged. No feedback related to the response was given to the subjects.

### Time Bisection Task

**FIGURE 2:**
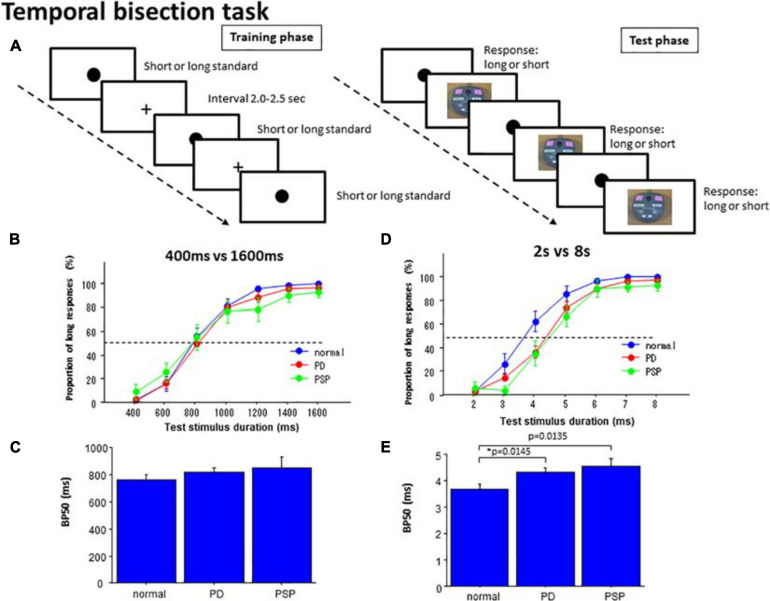
Temporal bisection task. **(A)** In the training phase, subjects were presented with standard durations of short and long durations until they learned the durations. In the test phase, subjects were required to judge whether the presented test duration was closer to the shorter or longer standard durations. **(B)** In the 400 versus 1600 ms task, the proportion of test trials in which subjects responded “long” was plotted as a function of test stimulus duration for each subject group (normal subjects, PD patients, and PSP patients). Error bars give standard errors. **(C)** Comparison of the bisection point at which subjects responded “long” with a probability of 50% (BP50) was compared among different subject groups in the 400 ms versus 1600 ms task. **(D)** A plot similar to B made for the 2 s versus 8 s bisection task. **(E)** A plot similar to **(C)** for the 2 s versus 8 s bisection task.

The time bisection task was to detect the “perceptual midpoint” (bisection point) of two standard stimulus durations (Figure 2A). The time bisection task consisted of two phases: training and testing (Merchant et al., 2011). In the training session, subjects were presented with two standard (anchor) stimulus durations (S1, S2), long or short (anchor duration), 20 times each to allow the subjects to become familiar with the time durations. For the presentation of shorter and longer anchors, a yellow-filled circle of 1.0 cm diameter appeared in the center of the monitor screen at a distance of 50 cm from the eyes of the subjects for the respective durations. The standard (anchor) durations were either 400 or 1600 ms in the short duration session (400 ms vs. 1600 ms task) and 2 and 8 s in the long duration session (2 s vs. 8 s task). The 1:4 ratio between the two standard (anchor) durations has been used in many previous studies (Merchant et al., 2011).

Subsequently, in the test phase, the subjects were consecutively presented with test stimuli of various durations, which had durations either equal to or intermediate between the two standard (anchor) durations. For test durations, visual stimuli of the same size and color with standard stimuli were presented. The duration varied in each trial between 400 and 1600 ms, with an increment of 200 ms during the short duration task, and from 2 to 8 s with an increment of 1 s during the long duration task. The durations were presented three times each in a pseudorandomized and counterbalanced manner. The subjects were to categorize them as short or long by pressing one of the two buttons (“long” or “short”), according to whether the duration of the test stimuli was judged to be closer to the shorter or longer of the standard (anchor) durations. Here again, accuracy of judgment rather than promptness of response was emphasized. No feedback was given to the subjects regarding the responses made.

### Time Production Task

**FIGURE 3:**
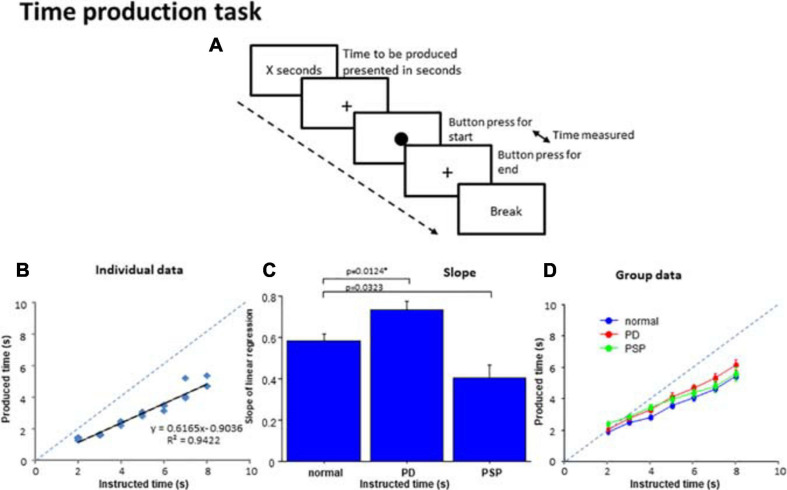
Time production task. **(A)** In the time production task, subjects were required to produce the time presented on the monitor screen in number of seconds. When the subjects press a button, a filled circle appears in the center of the screen. The subjects press the button again when they consider the time to be produced has elapsed, when the circle disappears and a cross reappears instead. The interval between the two button presses corresponds to the produced time. **(B)** The time produced was plotted as a function of the time instructed to produce for a normal subject. The dashed line indicates the line of unison on which the produced time equals the instructed time. **(C)** Comparison of the slopes of linear correlation among the three subject groups. **(D)** The time produced was plotted as a function of the time instructed to produce for the average of all subjects within each group. Error bars indicate standard errors. The dashed line indicates the line of unison as in **(B)**. Blue curve: normal subject, red curve: PD patients, green curve: PSP curve.

In the time production task, subjects were required to produce the time instructed in number of seconds (Figure 3A). This task required the subjects to make button presses to indicate the time produced, and included a motor component. At the beginning of the task, a number of seconds, such as “2 s” or “5 s,” appeared centrally on the monitor screen, placed approximately 50 cm in front of their eyes. This instructed the subjects on the time durations that they were required to produce. The instructed duration varied in each trial between 2 s and 8 s with 1 s increments, and appeared six times each during a session in a pseudorandom and counterbalanced order. Subjects were to produce the time duration by tapping twice on the space bar on the keyboard, that is, by creating an interval between them that would equal the instructed time duration. When the subjects pressed the button once, a filled circle of 1.0 cm diameter appeared centrally on the monitor screen. When they pressed the same button the second time, the circle disappeared, such that the interval between the two buttons presses corresponded to the duration of the circle appearing on the screen. Similarly to Honma et al. (2016), we allowed subjects to count to themselves in the supra-second tasks. This is what we do in real life if we want to “perceive” time in the supra-second range accurately; for perceiving or encoding durations of time longer than 2–3 s accurately, we need to “count” in bits, that is, at some time interval, whether implicitly or explicitly.

For the production of the instructed duration, the subjects were allowed to count silently to themselves, at their subjective sense of one second.

### Time Reproduction Task

**FIGURE 4:**
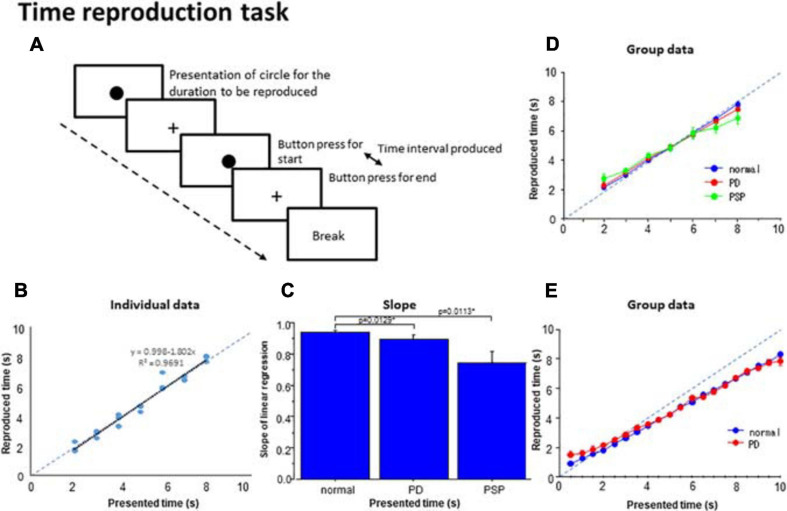
Time reproduction task. **(A)** Subjects were required to reproduce the time duration indicated by the presentation of a visual stimuli (circle) by making two button presses and making the interval between them equal to the presented duration. **(B)** The time reproduced was plotted as a function of the presented time duration in a subject. Dots represent data for individual trials. The dashed line indicates the line of unison over which the produced time equals the instructed time. **(C)** The slope of linear correlation was compared among the three subject groups. **(D)** A similar plot depicting the averaged group data. Error bars indicate standard errors. Dashed lines indicate the line of unison over which the produced time equals the instructed time. Blue curve: normal subjects, red curve: PD patients, green curve: PSP patients. **(E)** Subjects (normal subjects, PD patients) were required to reproduce the presented time duration ranging from 0.5 to 10 s similarly as above. Blue curve: normal subjects, red curve: PD patients.

In the time reproduction task, subjects were required to produce the duration presented (Figure 4A). In each trial, a circle of 1.5 cm diameter (instruction circle) first appeared on the monitor screen for a duration varying from 2 s to 8 s with 1 s increments and then disappeared. The duration of circle presentation indicated the time duration that subjects were to reproduce. Each duration appeared three times in a pseudorandom and counterbalanced order per session. After the presentation, similarly to the time production task, the subjects were to reproduce the instructed time duration by tapping twice on the space bar on the keyboard, that is, by creating an interval between them that would match the instructed time interval. When the subjects pressed the button the first time, a filled circle of 1.0 cm diameter, slightly smaller than the instruction circle, appeared on the monitor screen. When they pressed the same button the second time, the circle disappeared. In effect, the subjects made the circle appear on the monitor for the same duration as the instruction circle did. Again, similarly to Honma et al. (2016), for the reproduction of the instructed duration, the subjects were allowed to count silently to themselves when performing the task.

In a separate session, the reproduction task was performed similarly as described above but this time, the duration of the instruction circle presentation was randomly selected from among 20 possible durations (0.5, 1.0, 1.5 …, and 10.0 s) in 0.5 s increments.

### Data Analysis and Statistical Assessment

#### Duration Comparison/Bisection Tasks

In the duration comparison task, we calculated the proportion of trials with which the subjects responded that S2 duration was longer (“long” responses) and plotted this as a function of S2 duration, separately for each standard (S1) duration. Since this plot generally assumed a logistic-like curve as reported, curve fitting was made to a logistic function using commercial software (OriginPro2019, Lightstone, Tokyo, Japan). Goodness of fit was assessed by the *R*^2^ value adjusted for the degree of freedom. From the fitted curve, we derived the S2 duration corresponding to the 50% level where subjects were presumed to respond “long” with a 50% probability (point of subjective equality [PSE]). Also, as a measure of the temporal sensitivity, the difference limen (DL) was calculated. For this, we followed the standard procedure for building a control curve, e.g., to fit the measured response of substance response to varied concentrations of an agonist, which is implemented in OriginPro2019. The durations of presented stimuli to which the subjects responded “long” at 20 and 80% probability was derived from this fitted curve (P20 and P80). We calculated the DL by dividing the difference between these values by 2, namely (BP80 – BP20)/2.

In the time bisection task, similarly to the duration comparison task, for each duration presented, we calculated the proportion of trials in which the subjects responded that the test stimuli were closer in duration to the longer of the standard durations (“long” response). The proportion of “long” responses was plotted in the ordinate as a function of the test duration on the abscissa. Again, since the plot typically assumed a logistic-like curve, curve fitting was performed to the logistic function as above. Goodness of fit was evaluated by the *R*^2^ value adjusted for the degree of freedom. From the fitted curve, we derived the stimulus duration corresponding to the 50% level, that is, the duration of presented stimuli at which the subjects responded “long” or “short” with 50% probability, which was termed BP50. Based on data with a goodness of fit (adjusted *R*^2^ value) of curve fitting above 0.7, we derived the P50 value. DL was derived with a procedure same as that described above as an index of temporal sensitivity.

#### Production/Reproduction Tasks

For the production task, to assess the accuracy with which the subjects produced/reproduced the time intervals, we constructed a scatterplot for each subject with the indicated duration on the abscissa and the produced/reproduced duration on the ordinate. The slope was derived from the linear correlation, and the goodness of fit was calculated as the adjusted *R*^2^ value. Correlating the produced time with the indicated time interval, the slope of linear correlation would correspond to the length of time the subject perceives as 1 s, that is, the pace of the internal clock. A steeper slope would imply a slower internal clock while a shallower slope would imply a faster internal clock (see sections “Results” and “Discussion”).

In the reproduction task, a similar scattergram was constructed for each subject, plotting the produced/reproduced duration (the ordinate) as a function of the indicated duration (abscissa). Similar to the above, the slope of the regression line in the linear correlation was derived from this fitting, and the goodness of fit was derived as the adjusted *R*^2^ value. Since the reproduced time intervals averaged across all time intervals to be reproduced were overall similar (not statistically significantly different) across subject groups (see section “Results”), the shallower slope of the regression line would imply a larger migration effect. The migration effect causes the short time intervals to be reproduced longer and the long time intervals to be reproduced shorter, with the reproduced time intervals migrating toward the mean of all presented times, hence the shallower slope (see section “Discussion” for the migration effect). Conversely, a steeper slope implies a smaller migration effect.

### Statistical Assessment

Statistical analyses, including repeated measures analysis of variance (rmANOVA) and *t*-tests, were conducted using a commercial software package, SPSS statistics 17.0.0 (SPSS Japan, Inc., Tokyo).

### Duration Comparison/Temporal Bisection Tasks

For the duration comparison task, the proportion of button presses responding “long” was subjected to rmANOVA, with subject group as a between-subject factor (two levels, PD and normal subjects) and S2 duration (12 levels) as a within-subject factor, separately for the 700, 2100, and 3500 ms standard duration blocks. Also, PSE and DL was subjected to one-factor rmANOVA, with the factor subject group (two levels, PD and normal subjects). For *post hoc* analysis of this task and other tasks, we corrected for multiple comparisons using Bonferroni’s method. The significance level was *p* < 0.05. The effect size was assessed by partial eta squared (η_*p*_^2^, also for the following rmANOVA analyses).

For the bisection task, the proportion of button presses responding “long” was subjected to rmANOVA, with subject group as a between-subject factor (two levels, PD patients and normal subjects or three levels, PD, PSP patients, and normal subjects) and test stimulus duration (seven levels) as a within-subject factor, separately for the 700, 2100, and 3500 ms standard duration blocks. Also, BP50 and DL were subjected to one-factor rmANOVA, with factor subject group (two levels, PD and normal subjects).

To see how the clinical variables of patients influenced the performances in the perceptual timing tasks, multiple linear regression analyses were conducted in PD and PSP patients, separately (Table 3). We took the PSE and DL values in the duration comparison task and the BP50 and DL in the time bisection task as outcome variables, and the age of subjects, duration of disease, MMSE score, average SBR score, and H/M ratio as predictor variables in PD and PSP patients. The coefficient of determination was expressed as *R*^2^, and the partial regression coefficients of predictor variables were expressed as β. The *p*-value from the *t*-test for the regression slope of predictor variables was used to determine the probability of the analysis. For all analyses, the statistical significance criterion was set at *p* < 0.05.

**TABLE 3:** Results of the multiple linear regression analyses.

Variable	BP50 (400 ms vs. 800 ms) *R*^2^ = 0.478		BP50 (2 s vs. 8 s) *R*^2^ = 0.444		Slope (production) *R*^2^ = 0.444		Slope (reproduction) *R*^2^ = 0.360	
	*P*	β	*P*	β	*P*	β	*P*	β
Age	0.1575	–0.329	0.055	–0.408	0.0209*	–0.56	0.4408	–0.172
Duration	0.0115*	0.644	0.5955	–0.106	0.3592	0.195	0.4729	0.147
MMSE	0.4175	–0.18	0.0167*	–0.521	0.907	–0.025	0.0279*	0.511
Average SBR	0.7159	0.089	0.9813	0.005	0.2822	0.241	0.832	0.046
H/M ratio	0.7716	–0.07	0.0521	–0.42	0.8416	0.042	0.9454	0.015

*Significance at *p* < 0.05.

### Production/Reproduction Tasks

In the production task, two factors rmANOVA was performed on the produced time duration, with the subject group as a between-subject duration factor (PD and PSP patients and normal subjects, three levels) and instructed time as a within-subject factor (seven levels). For individual subjects, the slope of the regression line between the instructed and produced durations was derived. One-factor rmANOVA was performed on the slope of the regression line, with factor subject group. For the reproduction task, rmANOVA was similarly performed on the reproduced duration and the slope of the regression line.

To see how the clinical variables of patients influenced performance in the time production/reproduction tasks, multiple linear regression analyses were carried out on the slope values of time production/reproduction tasks in PD patients for whom the predictor variables below were available in sufficient number (Table 3). We took the slope of the linear correlation in the time production/reproduction tasks as the outcome variable, and the age of subjects, duration of disease, UPDRS motor score, MMSE score, average SBR score, and MIBG uptake score (H/M ratio) as predictor variables. UPDRS motor score was excluded from the variables, however, since the UPDRS motor score and average SBR score, both representing dopamine deficiency, are known to correlate significantly with each other (Pirker, 2003; Ikeda et al., 2019), and also showed a significant correlation in PD patients in the present study (*r* = –0.469, *p* = 0.0267). The coefficient of determination was expressed as adjusted *R*^2^, and the standardized partial regression coefficients of predictor variables were expressed as β.

## Results

### Duration Comparison Task

In this task, the subjects (12 PD patients and 15 normal subjects) compared the durations of two successive tones (S1, S2) presented successively. At the group level, when we plotted the proportion of “long” responses across subjects as a function of S2 duration for data (Figure 1A), the proportion of “long” responses reliably increased with the comparison tone duration across all standard durations, assuming a logistic-like curve (statistical results summarized in Table 4).

**TABLE 4:** Statistical results for the timing tasks.

Duration comparison task									
**Variable**	**Group**			**Duration**			**Duration X Group**		
**Proportion of long responses**	***F*_1,25_**	***p***	**η_*p*_^2^**	***F*_10,250_**	***p***	**η_*p*_^2^**	***F*_10,250_**	***p***	**η_*p*_^2^**

S1 = 700 ms	*F* = 4.981	*p* = 0.0348*	0.169	*F* = 100.79	*p* < 0.0001**	0.858	*F* = 2.178	*p* = 0.0197*	0.079
S1 = 2100 ms	*F* = 3.248	*p* = 0.0841	0.125	*F* = 96.075	*p* < 0.0001**	0.852	*F* = 1.921	*p* = 0.0431*	0.083
S1 = 3500 ms	*F* = 0.181	*p* = 0.6746	0.005	*F* = 95.157	*p* < 0.0001**	0.864	*F* = 0.686	*p* = 0.7367	0.026

**Temporal bisection task**									
**Variable**	**Group**			**Duration**			**Duration × Group**		
**Proportion long response**	***F*_2,126_**	***p***	**η_*p*_^2^**	***F*_6,378_**	***p***	**η_*p*_^2^**	***F*_12,756_**	***p***	**η_*p*_^2^**

400 ms vs. 1600 ms	*F* = 0.500	*p* = 0.6091	0.014	*F* = 206.014	*p* < 0.0001**	0.695	*F* = 0.974	*p* = 0.4729	0.034
2 s vs. 8 s	*F* = 6.221	*p* = 0.0035*	0.183	*F* = 197.270	*p* < 0.0001**	0.766	*F* = 1.347	*p* = 0.1894	0.063

**Time production task**									
**Variable**	**Group**			**Duration**			**Duration × Group**		
**Produced time**	***F*_2,126_**	***p***	**η_*p*_^2^**	***F*_6,378_**	***p***	**η_*p*_^2^**	***F*_12,756_**	***p***	**η_*p*_^2^**

	*F* = 1.360	*p* = 0.2638	0.040	*F* = 269.251	*p* < 0.0001**	0.806	*F* = 2.118	*p* = 0.0151*	0.061

**Time reproduction task**									
**Variable**	**Group**			**Duration**			**Duration × Group**		
**Reproduced time**	***F*_2,126_**	***p***	**η_*p*_^2^**	***F*_6,378_**	***p***	**η_*p*_^2^**	***F*_12,756_**	***P***	**η_*p*_^2^**

	*F* = 0.028	*p* = 0.9724	0.001	*F* = 573.619	*p* < 0.0001**	0.910	*F* = 3.716	*p* < 0.0001	0.115

*Significance at *p* < 0.05. **Significance at *p* < 0.0001.

In the 700 ms standard duration block (Figure 1B and Table 4), the effect of group was significant, whereas the time × group interaction was also significant. This reflected the fact that the proportion of “long” responses was larger in PD patients; patients evaluated the S2 duration as longer in duration than normal subjects, and also compared with the veridical value. There was a leftward shift of the logistic curve in PD patients relative to normal subjects. Also at the individual level, when we plotted the proportion of “long” responses against the S2 duration, this assumed a logistic-like curve. The curve for individual subjects fitted fairly well with the logistic function (adjusted *R*^2^ value: PD 0.8939 ± 0.0855, normal subjects 0.9115 ± 0.1159). The PSE derived from the individual curve was significantly smaller in PD patients than in normal subjects [Figure 1C; normal subjects 814.85 ± 26.85 ms, PD 736.51 ± 24.21 ms, *F*(1,25) = 4.470, *p* = 0.0446, η_*p*_^2^ = 0.152], suggesting an overestimation of S2 duration. DL did not differ statistically between the two groups [*F*(1,25) = 0.414, *p* = 0.5258, η_*p*_^2^ = 0.016].

In the 2100 ms standard duration block (Figure 1D and Table 4), the proportion of “long” responses increased reliably with S2 duration. The effect of group showed a trend that just failed to reach significance, whereas the time × group interaction was significant. This reflected the fact that the proportion of “long” responses was slightly but significantly larger in PD patients than in normal subjects, especially at intermediate S2 durations; there was a leftward shift of the logistic curve in PD patients relative to normal subjects. The PSE value derived from this curve was significantly smaller for PD subjects than for normal subjects [Figure 1E and Table 4; normal subjects 2224.84 ± 60.07 ms, PD 2006.39 ± 80.70 ms, effect of group: *F*(1,25) = 4.917, *p* = 0.0359, η_*p*_^2^ = 0.164]; there was overestimation of S2 duration. DL was not statistically different between the two groups [*F*(1,25) = 2.636, *p* = 0.1170, η_*p*_^2^ = 0.095].

In contrast, in the 3500 ms standard duration block (Figure 1F and Table 4), the effect of time was significant, whereas the effect of group was not significant. The time × group interaction also failed to reach significance; there was no significant difference in the proportion of “long” responses between PD patients and normal subjects. Again, at the individual level, we plotted the proportion of “long” responses against the S2 duration, and performed curve fitting with a logistic function. Neither PSE [Figure 1G; normal subjects 3353.75 ± 62.86 ms, PD 3444.18 ± 105.94 ms, *F*(1,25) = 0.590, *p* = 0.4498, η_*p*_^2^ = 0.023] nor DL differed between the two groups [*F*(1,25) = 1.244, *p* = 0.275, η_*p*_^2^ = 0.047].

### Temporal Bisection Task

#### 400 ms vs. 1600 ms Task

In the 400 and 1600 ms bisection task, the proportion of responses responding “long” was averaged across subjects. This was plotted as a function of the test stimulus duration in the individual subject for PD, PSP, and normal subjects (NC; Figure 2B and Table 4). As reported previously in human and animal studies (Droit-Volet et al., 2007), the proportion of “long” responses systematically increased with test duration. There was no difference in the proportion of “long” responses at any of the test stimulus durations among the three groups of subjects (Table 4). This corroborated the visual inspection above that both curves for the PD and PSP patients increased with increasing test duration and largely overlapped with that of normal subjects; there was no significant difference in performances for the 400 ms vs. 1600 ms bisection task among the three subject groups (NC, PD, and PSP).

At the individual level, the proportion of “long” responses as a function of test stimulus duration assumed a logistic-like curve. In fact, the data of each individual subject fitted fairly well with a logistic function in normal subjects (adjusted *R*^2^: 0.776 ± 0.095), but less well in PD patients (adjusted *R*^2^: PD 0.626 ± 0.279, difference from normal subjects failed to reach significance at *p* = 0.0485 corrected for multiple comparisons) and PSP patients (PSP: 0.547 ± 0.307, significant difference comparison between PD vs. normal at 4 s: *p* = 0.0456, PSP vs. normal: *p* = 0.0124, PD vs. PSP: *p* = 0.3332, where the cutoff value with correction for multiple comparisons was *p* = 0.0167). BP50 derived from the logistic function was not significantly different in PD and PSP patients relative to normal subjects [Figure 2C; effect of group: *F*(2,126) = 0.824, *p* = 0.4439, η_*p*_^2^ = 0.026]. The DL did not differ significantly among the three groups [effect of group: *F*(2,126) = 0.787, *p* = 0.4598, η_*p*_^2^ = 0.028].

In PD patients, multiple regression analysis showed no subject factors that showed a significant correlation with BP50. This was also true for PSP patients (Table 3).

#### 2 s vs. 8 s Task

In the 2 s vs. 8 s bisection task, the curve plotting the proportion of “long” responses averaged across subjects as a function of the test stimulus duration again assumed a logistic-like function (Figure 2D). rmANOVA found a significant effect of group and test stimulus duration, whereas the interaction between the two factors was not significant. *Post hoc* analysis showed that PD and PSP patients responded “long” in a significantly smaller proportion of trials relative to normal subjects whereas PD and PSP patients were not statistically different (comparison between PD vs. normal at 4 s: *p* = 0.0009, PSP vs. normal: *p* = 0.001, PD vs. PSP: *p* = 0.5138, where the cutoff value with correction for multiple comparisons was *p* = 0.0167). PSP was also significantly different from normal subjects at 5 s (*post hoc* analysis: *p* = 0.0019). This indicated a rightward shift of the logistic curve for both PD and PSP patients relative to normal subjects.

Also at the individual level, the graph plotting the proportion of responses responding “long” as a function of the test stimulus duration assumed a logistic-like curve. Indeed, curve fitting showed a fair correlation with logistic function individually in normal subjects (adjusted *R*^2^: normal subjects 0.735 ± 0.175). Although the goodness of fit was smaller for PD patients (0.644 ± 0.264, difference from normal subjects at *p* = 0.0445 corrected for multiple comparisons, Cohen’s *d* = 0.406) and PSP patients relative to normal subjects (0.585 ± 0.299, *d* = 0.612), the difference among the three subject groups was not significant [effect of group: *F*(2,126) = 1.710, *p* = 0.1896, η_*p*_^2^ = 0.048].

ANOVA performed on BP50, derived from the curve fitting in individual subjects, revealed a significant effect of group [Figure 2E; *F*(2,126) = 4.243, *p* = 0.0191, η_*p*_^2^ = 0.128], indicating significantly larger BP50 in PD and PSP patients than that of normal subjects (*p* = 0.0145, Cohen’s *d* = 0.768; *p* = 0.0135, *d* = 1.054 after correction for multiple comparisons whereas the cutoff value for multiple comparisons was *p* = 0.0167; PD and PSP patients were not statistically different: *p* = 0.4594, η_*p*_^2^ = 0.150). DL did not differ significantly among the three subject groups [effect of group: *F*(2,126) = 0.854, *p* = 0.4307, η_*p*_^2^ = 0.048]. Again, these effectively suggested the rightward shift of the logistic curve for PD and PSP patients relative to normal subjects.

In PD patients, multiple regression analysis showed that, among the subject factors, only the MMSE score showed a significant negative correlation with BP50 (*p* = 0.0167, β = –0.521), accounting for 43.4% of the variance (Table 3). In PSP, none of the four factors contributed significantly to BP50, which may be due to the variability of data and small number of subjects.

### Time Production Task

For each subject, a scatterplot was constructed plotting the produced duration as a function of the duration indicated in number of seconds (Figure 3B). Generally, the linear correlation between the instructed and produced durations was fairly good at the individual level for normal and PD groups, but was less good in PSP patients. The adjusted *R*^2^ was significantly smaller on average in PSP patients compared to normal subjects and PD patients, reflecting the variability of performance in this group [adjusted *R*^2^: normal subjects 0.913 ± 0.075, PD 0.917 ± 0.068, PSP 0.741 ± 0.210; *F*(2,126) = 5.029, *p* < 0.0001; *post hoc* analysis revealed a significant difference between PSP patients and normal subjects and PD patients; normal vs. PSP: *p* < 0.0001, Cohen’s *d* = 1.386, and PD vs. PSP: *p* < 0.0001, *d* = 1.470; PD patients and normal subjects were not statistically different: *p* = 0.9112, *d* = 0.172].

The slope was derived from the linear correlation in individual subjects. When rmANOVA was conducted for the slope of the linear correlation, the effect of group was significant [*F*(2,126) = 10.797, *p* < 0.0001, η*_*p*_^2^* = 0.261], which reflected the fact that PD patients showed a slightly but significantly steeper slope (*p* = 0.0124 after correction for multiple comparison; cutoff value for multiple comparisons: *p* = 0.0167 Cohen’s *d* = 0.777) whereas PSP patients showed a trend for a shallower slope compared with normal subjects (*p* = 0.0323 after correction for multiple comparison, *d* = 1.035) (Figure 3C). This indicated that the “subjective sense of 1 s” was longer for PD patients and shorter for PSP patients than normal subjects. In comparison with the line of unison, however, the produced time was shorter than veridical in all groups.

At the group level, we performed rmANOVA on produced time averaged across subjects, as a function of the indicated duration (Figure 3D and Table 4). The reproduced duration increased reliably with the indicated time, although, on average, there were no significant overall differences in produced time among PD, PSP patients, and normal subjects. There was, however, a significant interaction between group and presented duration. This suggested that the slope, that is, the increase of produced duration per unit increased duration indicated, was smaller for PSP patients and slightly larger for PD patients in comparison with normal subjects, consistent with the results shown in Figure 3B. If we postulate that the slope of this correlation indicates the approximate subjective sense of 1 s in each subject (see section “Discussion”), PD patients would be considered to exhibit a slower pace of the internal clock, that is, a longer internal sense of 1 s whereas PSP patients showed a faster internal clock compared with normal subjects (see section “Discussion”).

In PD patients, multiple regression analysis showed that, among the subject factors, the slope of the regression line showed a significant negative correlation only with age (β = –0.560, *p* = 0.0209). With increasing age, the slope became shallower, that is, the internal clock became faster (Table 3). Age accounted for 76.0% of the total variance. The average SBR score, indicating the degree of dopamine deficiency, did not show a significant contribution to the slope. In PSP, none of the four factors contributed significantly to BP50, which may be due to the variability of data in these subjects.

### Time Reproduction Task

At the individual level, we plotted the reproduced duration against the presented duration. The reproduced duration showed a good linear correlation with the presented duration in normal subjects and PD patients (Figure 4B; adjusted *R*^2^: normal subjects 0.933 ± 0.012, PD 0.870 ± 0.027), whereas the goodness of fit was significantly smaller for PSP patients (0.726 ± 0.084). *Post hoc* analysis revealed that the adjusted *R*^2^ was significantly smaller for PSP patients compared with the other two groups whereas the latter two groups were not statistically different [effect of group: *F*(2,126) = 6.136, *p* = 0.0039, η_*p*_^2^ = 0.177; normal subject vs. PSP: *p* = 0.0039, Cohen’s *d* = 1.141, PD vs. PSP: *p* = 0.0125, *d* = 0.695 corrected for multiple comparisons, normal subjects vs. PD: *p* = 0.3324 corrected for multiple comparisons; cutoff value: *p* = 0.0167, *d* = 0.556].

The slope of the linear correlation differed significantly across subject groups [effect of group: *F*(2,126) = 5.746, *p* = 0.0052, η*_*p*_^2^* = 0.243]. Consistent with the above results, the slope of linear correlation was slightly shallower in PD patients and significantly more shallower in PSP patients relative to normal subjects (Figure 4C; normal subjects 0.958 ± 0.013; PD patients: 0.858 ± 0.026; PSP patients: 0.726 ± 0.069). *Post hoc* analysis showed a significantly shallower slope in PSP patients compared to normal subjects (*p* < 0.0001 after correction for multiple comparison, Cohen’s *d* = 1.369) and PD patients (*p* = 0.0113, *d* = 0.767 after correction for multiple comparisons; cutoff value: *p* = 0.0167 after correction for multiple comparisons).

In PD patients, multiple regression analysis revealed that, among the clinical variables, the slope of the regression line showed a trend for positive correlation only with MMSE score (β = 0.511, *p* = 0.0279), which accounted for 82.9% of the total variance (Table 3). Other variables did not contribute significantly to the slope of correlation. For PSP patients, multiple regression analysis did not show any factors that significantly contributed to the slope of the regression line.

Figure 4D shows the reproduced duration as a function of the presented duration at the group level, plotted separately for the three subject groups. rmANOVA conducted on the reproduced duration showed that there was a significant effect of time (Table 4), suggesting that reproduced duration increased reliably with the presented duration for all groups. Although there was no significant effect of group, there was a significant interaction between group and presented duration. This indicated that while the overall reproduced duration was similar, on average, across subject groups, patients, especially PSP patients, over-reproduced shorter time durations than this range and under-reproduced durations longer than this. Intermediate durations were reproduced approximately equal to the presented duration in all subject groups; that is, a 5 s time interval was fairly accurately reproduced as 5 s. This trend was much more evident in PSP patients, and was very mild for PD patients. This significant interaction persisted when we compared PD patients and normal subjects only [effect of time: *F*(6,324) = 827.839, *p* < 0.0001, η_*p*_^2^ = 0.948; effect of group: *F*(1,54) = 0.040, *p* = 0.8424, η_*p*_^2^ = 0.001; time × group: *F*(6,324) = 2.605, *p* = 0.018, η_*p*_^2^ = 0.0585], reflecting the fact that PD patients exhibited a slightly shallower slope relative to normal subjects.

Twelve PD patients and 14 normal subjects performed a similar reproduction task in which the durations to be reproduced ranged from 0.5 to 10 s. Figure 4E shows that the shorter presented durations were reproduced as longer than the presented duration while longer presented durations were reproduced as shorter than the presented time in the patient groups. As a consequence, the slopes for PD patients were shallower than those for normal subjects. Two-factor factorial ANOVA revealed a significant effect of presented duration [*F*(19,456) = 539.222, *p* < 0.0001, η_*p*_^2^ = 0.804], indicating that the reproduced duration increased reliably with the presented duration. The effect of subject group was also significant [*F*(1,24) = 5.471, *p* = 0.0194, η_*p*_^2^ = 0.02], with a significant interaction between group and presented duration [*F*(19,456) = 1.595, *p* = 0.0490, η_*p*_^2^ = 0.012]. This corroborated Figure 4E, which shows that the reproduced durations of PD patients were slightly longer than that of healthy volunteers when the presented duration was under 4 s, whereas both PD patients and normal subjects under-reproduced presented time intervals above 4 s to a similar degree. A *post hoc* test failed to show a significant difference between these groups at the presented time above 4 s (*p* > 0.1), except at the presented duration of 10 s where PD patients significantly under-reproduced the time duration compared to the veridical 10 s (*p* = 0.0171). Also for the shorter presented durations of 0.5–2 s, the difference became significant when the data between presented times of 0.5–2 s was collapsed and compared between PD patients and normal subjects (*p* = 0.0001 Cohen’s *d* = 0.258).

## Discussion

In the present study, we characterized the temporal sensation of PD using perceptual and motor timing tasks. Short and long time durations were studied: durations under 2 s (corresponding to the “sub-second” range) and those above 2 s (corresponding to the “supra-second” range). Time distortion was noted in both the supra- and sub-second ranges, not only in the reproduction task as reported previously (Malapani et al., 1998, 2002) but also in other timing tasks. We will discuss the overall pattern of results in the context of SET by first referring to whether the subjective sense of 1 s or the “pace of the internal clock” was slowed in PD patients.

### Production Task -Is the Subjective Sense of 1 s or the Pace of the “Internal Clock” Slowed in PD Patients?

In the production task, the slope of the regression line between the instructed and the actually produced time duration was presumed to provide an index of the pace of the internal clock corresponding to the “subjective sense” of 1 s, since no feedback was given to the subjects. The slope was slightly but significantly steeper in PD patients than in normal subjects, suggesting a slower pace of the internal clock than normal, consistent with studies reporting underestimation of time (Pastor et al., 1992a; Lange et al., 1995) and overproduction of time (Lange et al., 1995; Jones et al., 2008). Since we allowed subjects to count silently during the task, the overproduced time duration corresponding to the subjective 1 s could have resulted partially from the slowness of implicit counting, although Jones et al. (2008) reported that overestimation of time production occurs even when PD patient were explicitly told not to count.

The slope of the regression line correlated significantly with age, but not with the average SBR score, implying that dopamine deficiency contributed only mildly at best to the slowed pace of the internal clock. Similarly, studies of temporal perceptual performance in PD have shown no impairment (Riesen and Schnider, 2001; Perbal et al., 2005; Wearden et al., 2008; Wild-Wall et al., 2008), faster-paced internal clock for producing time intervals above 10 s (Wojtecki et al., 2011; Honma et al., 2016, 2017), or no significant associations with clinical ratings of disease severity (Ivry and Keele, 1989; Artieda et al., 1992; Rammsayer and Classen, 1997; Breitenstein et al., 2001; Smith et al., 2007). Since we studied PD patients 3–4 h after their last drug intake, this slight difference may be due persistent effect of dopaminergic medication partially normalizing the magnitude of abnormality.

Additionally, it should be noted that this slowed subjective sense of 1 s cannot be extrapolated to the subjective sense of longer time intervals. As the time intervals to be produced became longer (over 2–3 s), they were generally produced shorter than the instructed time duration (underproduction), a pattern that was also observed in normal subjects (Glicksohn and Leshem, 2010). The magnitude of this underestimation was much more prominent compared to the relatively small difference in the linear correlation slopes among the three subject groups (Figure 3).

Remarkably, the underproduction of longer time intervals occurred even though the subjects were allowed to consciously match each count of every 1 s with their subjective sense of 1 s by counting. The underproduction thus suggests that the pace of the internal clock changed from fast to slow as counting went on, without the subjects being aware of it. A similar pattern of over-reproduction of shorter time intervals and over-reproduction of longer time intervals was noted by Honma et al. (2016), who asked normal subjects and PD patients on medication to keep tapping at their subjective interval of 1 s up to a period of 100 s. Relative to normal subjects, PD patients over-reproduced the shorter time intervals below 5 s whereas they under-reproduced longer time intervals equal to or above 5 s. Contrasting time production and reproduction tasks, they interpreted this dysfunctional time processing as occurring independent of working memory ability. This shortening of the time span may be likened to hastening in PD; patients tend to speed up during unpaced, repetitive finger or lip movements (Ackermann et al., 1997; Konczak et al., 1997).

Pace of the internal clock can be studied by the synchronized tapping task, which requires subjects to press a button or to tap a keyboard in synchrony with repetitive tones presented at fixed interstimulus intervals (ISIs) (synchronization task, S) and, subsequently, to continue tapping at the same pace even after the tones have been removed (continuation task, C). The tapping pace in the S-C task is inconsistent, and has been reported to be either faster (Ivry and Keele, 1989; O’Boyle et al., 1996; Harrington et al., 1998a; Jones et al., 2011), slower (Pastor et al., 1992a), or unimpaired (Duchek et al., 1994; Spencer and Ivry, 2005; Wojtecki et al., 2011; Joundi et al., 2012) relative to normal subjects. Since the performance of tapping depends on the ISI used, Tokushige et al. (2018) performed a synchronized tapping task using various fixed ISIs between 200 and 4800 ms in separate blocks to study how fast and slow a rhythm subjects can synchronize with in order to assess the pace of their internal clock; when PD patients in the ON state were asked to tap in synchrony with tones presented at regular intervals (ISIs) of up to 2–3 s, early PD patients tend to tap slightly ahead of the tones (negative asynchrony). Although also noted in normal subjects, negative asynchrony is reported to be even more prominent in PD patients than in normal subjects (Diedrichsen et al., 2003), which became more apparent as time passed. This suggested a faster ticking of the internal clock in the early stages of PD, even in the presence of pacing tones. This was likened to hastening, which is often observed in PD patients when they are engaged in repetitive tapping, or in PD patients with freezing of gait (Tolleson C.M. et al., 2015). In later stages of PD, the hastening disappeared. Also, PD patients on medication who were asked to produce a time duration of 10 s, produced it shorter than the veridical value at around 8 s whereas normal subjects produced it almost veridically (Honma et al., 2017).

Judging from the slope of linear correlation, the subjective sense of 1 s in PSP patients was shorter, that is, they had a faster internal clock. Since our PD and PSP patients showed similar UPDRS motor and SBR scores, the difference for PD cannot be accounted for by the magnitude of dopaminergic deficiency alone, but may also be due to dysfunction of other neural systems pivotal for timing performance, such as the frontal lobe for short-term memory (Wild-Wall et al., 2008). Although this task involved a minimal memory component, it is possible that the temporal representation in the mind would have decayed during the course of production (Honma et al., 2018), leading to a shorter 1 s interval as time production proceeded, or that a phenomenon similar to motor hastening occurred.

### Bisection Task -Conscious and Subconscious Perception of Sub- and Supra-Second Time

The bisection task was mainly a perceptual task since only accurate judgment for classifying the test duration into “long” and “short” was required (and not prompt motor responses). In the bisection task with standard durations of 400 and 1600 ms, there was no difference in performance between normal subjects and PD patients. In contrast, for standard durations of 2 and 8 s, a rightward shift was noted in the logistic-like curve plotting the proportion of “long” responses as a function of the test duration, as reflected in the increased P50 value.

Animal studies show that dopamine deficiency induced by dopamine D2 blockers such as haloperidol causes a proportional rightward shift in the psychophysical function of the bisection task. Consistent with our results, intermediate longer durations in a set are less likely to be classified as ‘long,’ consistent with the slowed clock hypothesis (Meck, 1986, 1998, 2006; MacDonald and Meck, 2004, 2005, 2006). This cannot, however, explain why the shift occurred only for the 2 s vs. 8 s but not for the 400 ms vs. 1600 ms bisection task. Furthermore, a rightward shift was also noted in PSP patients. In contrast, the production task suggested a slower internal clock pace for PD but a faster pace for PSP patients.

When performing this task, the subjects may have to store the durations corresponding to the bisection point in reference memory and sort the test durations into “long” or “short” categories relative to this time scale (Allan and Gibbon, 1991; Matthews and Meck, 2016). If the time scale becomes longer, test durations would be judged as relatively shorter, causing its underestimation (namely, an increase in P50). Thus, the supra-second time scale may have undergone shortening while being retrieved from memory and compared with test durations whereas the sub-second time scale was unchanged. In our PD patients, the MMSE score showed a significant correlation with short-term memory, suggesting that the performance is related to the time scale stored in the short-term memory. There are studies suggesting that sub- and supra-second-range perceptual timing tasks in PD are impaired (Smith et al., 2007; Merchant et al., 2008a) or unimpaired (Wearden et al., 2008). Although the supra-second range has never been studied in the temporal bisection task in human PD patients, the results were consistent with the results of the animal studies of dopamine deficiency by Ward and Odum (2006). The difference between ranges may be consistent with the supra- and sub-second dichotomy raised in the section “Introduction.”

An alternative possibility is that the change in the bisection point arose while they were perceived/encoded during the training phase. In the 2 s vs. 8 s bisection task, if the standard durations are perceived by the pace of an internal clock that becomes faster as time goes by (as discussed above), the longer of the standard durations would be perceived (counted) as longer than veridical (e.g., 8 s standard perceived as being 10 s in duration) while the perception of the 2 s standard duration would not be dissociated largely from its veridical value. This would cause the perceived “bisection point” to shift toward a longer range. Consequently, test durations compared with this lengthened time scale would be judged as relatively shorter, leading to a rightward shift in the logistic-like function. With 400 ms vs. 1600 ms standards, this shift in bisection point was minimal, since the pace of the clock would change little during the brief standard durations.

### Duration Comparison Task

Several studies have addressed duration discrimination tasks mostly in the sub-second range, either reporting impairment (Rammsayer and Classen, 1997; Harrington et al., 1998a, 2011; Riesen and Schnider, 2001; Guehl et al., 2008) or no impairment in PD (Ivry and Keele, 1989; Hellstrom et al., 1997; Wojtecki et al., 2011). Here, we have shown in the temporal comparison task that for 700 and 2100 ms standards, relative overestimation of S2 duration occurred, that is, the subjects judged S2 (which has a shorter duration than S1) to be equal in duration to S1, but not for the 3500 ms standard.

If we postulate that the “sensory” trace of S1 duration persists to be directly matched with S2 duration in this task, without involving short-term memory, S2 duration perceived would be approximately identical to S1 duration, regardless of the standard duration. The results showed that S2 duration was overestimated relative to S1 duration, suggesting that the change of the “sensory memory” during the delay period of 2–2.5 s between the two stimuli. Wearden et al. (2008) found that the only task that significantly discriminated PD patients from the control group was a duration discrimination task that required the standard duration to be held in memory for 2, 4, and 8 s, but not when the delay was short (1.1 s). On the other hand, the 3500 ms standard duration may have been too long to be perceived “automatically” as a perceptual unit, and processing for this duration may differ from those of sub-second durations (see above for the sub- and supra-second dichotomy).

Jahanshahi et al. (2006), Harrington et al. (2011), and Jones and Jahanshahi (2014b) scanned PD patients on and off dopaminergic medication and normal subjects, while they performed a duration discrimination (comparison) task, with comparison durations separated by a delay period of several seconds. Greater striatal hypoactivation was found in both phases in PD than in normal subjects, suggesting not only the important role of the striatum in timing, but also cortical network engaged in working memory, including the middle frontal-inferior parietal regions and parahippocampal gyri. The MMSE score also correlated to P50 in our PD patients.

Alternatively, a mechanism similar to the duration adaptation can also explain the overestimation of S2 duration. The second duration is perceived as shorter when the same time duration is repeatedly presented at short intervals, which disappears when the interval gets longer (Matthews, 2011; Matthews and Meck, 2016). After repetitive exposure to stimuli of relatively short duration, a subsequent test stimulus of long duration is perceived as longer whereas after exposure to stimuli of relatively long duration, a subsequent test stimulus of short duration is perceived as shorter, for both sub- and supra-second time domains (Shima et al., 2016). But the possibility regarding adaptation is unlikely since it is demonstrated that presenting one stimulus (adaptor) does not produce any aftereffects (Li et al., 2017).

### Time Reproduction Task

Theoretically, measuring the presented time span by counting at a certain pace and reproducing it by counting at the same pace, subjects would reproduce the approximate time duration accurately as the presented regardless of the “pace” of the clock. However, Figure 4D shows that the subjects over-reproduced shorter time intervals and under-reproduced longer time intervals, whereas time intervals in between (around 4–5 s) were reproduced approximately the same as normal subjects; overproduction and underproduction were evident at “extremes” of the time range studied, similarly to previous reports of time reproduction tasks (Malapani et al., 1998, 2002; Koch et al., 2008): when PD patients learn and reproduce two target intervals (6 s, 18 s) in the same session while off medication, they overestimate shorter durations and underestimate longer durations in the seconds range, which is corrected by dopaminergic treatment (migration effect; Malapani et al., 1998, 2002).

Models have been proposed that postulate an accumulator receiving inputs from a pacemaker periodically emitting certain pulses per unit time corresponding to the clock pace (Hopson, 2003; Malapani and Ratikin, 2003; Shea-Brown et al., 2006). The accumulator in the models consists of an assembly of neural nodes that can be either in an ON or OFF state. Each pulse of the pacemaker would turn the OFF units into ON with a certain probability (gain), which then stays on. Transition from OFF to ON can also occur with a certain probability between the pacemaker pulses (decay). The gain and delay together determine how the total number of active accumulator units is accumulated with time, which corresponds to the subjective sense of time. In this simulation, the number of active nodes rises initially with time, but as real time goes by, the initial linear rise of accumulator nodes gradually decelerates, representing memory decay, assuming a curvilinear function of real time. It may even asymptote to a certain level at which the gain and decay is counterbalanced. If the curvilinear function for the temporal encoding temporal span into memory differs from that reading it out of from memory, more specifically, if it initially rises more rapidly and then decelerates earlier at a lower level for retrieving memory than for encoding time, this would result in a systematic error of timing and counting with underestimation of longer intervals and overestimation of shorter intervals as the encoded number of accumulator is read out from reproduction, leading to the “migration effect.” This may also explain why overproduction and underproduction is noted most prominently at “extremes” of the time ranges studied (Figure 4D).

Based on the finding that the migration effect does not occur when only one time duration is included in a single session, the shift of reproduced temporal duration has been ascribed to dysfunctional short-term memory (Malapani et al., 1998, 2002), more specifically, “temporal memory averaging,” when two or more temporal representations have to be stored in memory (Swanton et al., 2009; Kurti et al., 2014; Matell, 2014).

When the time intervals to be reproduced ranged from 0.5 to 10 s (Figure 4E), the degree of underproduction for longer presented time durations in the production task became more prominent than the overproduction of shorter time durations. Thus, the reproduction task is not totally consistent with the “averaging” effect. A similar finding has been reported by Honma et al. (2016), who asked normal subjects and PD patients to reproduce presented time intervals of 1–20 s by tapping at their subjective 1 s intervals.

Furthermore, it should be noted that counting was allowed when subjects performed this task. As a result, information on the presented time span would be retained verbally and prevent it from “decaying” or “getting mixed.” In both time production and time reproduction tasks, there was a general trend of overproducing shorter time durations and underproducing longer time durations. With the accelerating clock discussed above, if the subjects matched the duration with the pace of the accelerating internal clock, say every 1 s, the produced time would be progressively underproduced as time passes in the same way for PD patients and normal subjects.

When the produced/reproduced duration was plotted against the instructed time, this function showed a shallower slope than the line of unison. The crossing point between the plotted function and the line of unison took place at around 2–3 s. Taking the slope of linear correlation between the presented and reproduced duration as an index of the migration effect, the MMSE score showed a significant correlation with the slope in our PD patients, suggesting an association with short-term or working memory. A similar correlation between the degree of migration effect and cognitive dysfunction has been reported in patients with mild cognitive impairment (Dušek et al., 2012; Maaß et al., 2019).

The inaccuracy of reproduced duration in terms of the migration effect was even more pronounced in PSP patients, indicating inaccurate memory representation of time intervals. Frontal dysfunction, including that of the dorsalateral prefrontal cortex, may contribute to short-term memory dysfunction (Litvan et al., 1989; Litvan, 1994; Harrington et al., 1998b) and to a more prominent migration effect. In PSP patients, frontal dysfunction may be larger than in PD patients, leading to greater short-term memory dysfunction.

The limitation of the present study was the small number of trials. Although this was a necessary compromise in experimental design due to the time constraint of the experiment, the poor fitting performances as reflected in *R*^2^ values reported here may be due to this small number of trials, and the parameter estimates may not have been reliable enough. Secondly, although the experiments were done 3–4 h after the last drug intake, the persisting dopaminergic effect may have masked the possible changes in temporal performance of PD patients that may be ascribed to dopamine deficiency.

## Conclusion

Overall, the results cannot be explained simply by a monotonic linear relationship between the subjective experience of time and the passage of real time consistent with the slowed pacemaker hypothesis due to dopamine deficiency. Although it is difficult to draw a coherent picture encompassing the entire pattern of results, here we interpret the results by postulating a cognitive time scale or representation, especially for supra-second time, into which the perceived time is encoded in memory and from which memory is read out for later timing performance. We have seen that the time scale can vary depending on the task performed, which urge caution about using temporal production as an index of subjective perceptual duration (Matthews, 2011). The clock or time-keeping mechanism to monitor the progression of motor timing tasks (time production/reproduction) was considered to tick faster as time passes (an “accelerating” clock). Superimposed on top of this was a mild effect of the possibly “slowed” internal clock of PD patients due to dopamine deficiency. In the reproduction task, time distortion also occurred in short-term memory, exaggerated especially in PSP patients, such that short time durations were reproduced as longer and long durations were reproduced as shorter. Perceptual timing tasks showed changes apparently in the opposite directions to those observed in the motor timing tasks: underestimating shorter durations and overestimating longer durations. These distortions in time scale may occur through the very process of time production/reproduction (motor timing tasks), may be imposed by incoming prior sensory traces (perceptual timing tasks), get mixed in memory (reproduction tasks), and/or finally determined by the pathophysiology of PD. In this context, Honma et al. (2018, 2021) required PD patients to produce a subjective time interval of 10 s, and found that they tend to underproduce it, say, by about 2 s, i.e., they produced a time interval of 8 s on average, whereas normal subjects produced it closer to the veridical 10 s. PD patients were then given false feedback training in which they were trained with the veridical 10 s with feedback, by which the underproduction was temporarily corrected to be closer to the correct value (Honma et al., 2018, 2021). However, their subjective sense of time returned to their pre-training performances within a few minutes after the feedback was removed. Thus, the “subjective” time scale is robust within each subject, returning to its “inherent” value after feedback training in PD patients in a relatively short while. A similar “return” is noted even in normal subjects, but occurring much more gradually over several hours (Honma et al., 2021). The “inherent” time scale may be partly determined by the pathophysiology underlying PD, cognitively or unconsciously by prior experience, or imposed by instantaneous and incoming prior sensory traces. Whatever its origin, a traction toward the inherent time scale may further cause a change in the time scale during timing tasks, especially in the supra-second range. In contrast, normal subjects retained the learning for the same duration of time even after feedback was removed; their clock was more malleable. Timing performance in PD patients may actually be determined by such complex interactions among different time scales on the motor and sensory sides and memory, which may account for the controversial results in the timing task reported in PD so far.

## Data Availability Statement

The raw data supporting the conclusions of this article will be made available by the authors, without undue reservation.

## Ethics Statement

The studies involving human participants were reviewed and approved by the Faculty of Medicine Research Ethics Committee, Kyorin University, and the Jikei University Katsushika Medical Center. The patients/participants provided their written informed consent to participate in this study.

## Author Contributions

YT, MH, YU, and MS designed the experiments. YT, YA, S-iT, TF, TM, SI-T, AU, and SM carried out the collection and assessment of data. YT, MH, and YU carried out the study conception and design, data analysis and interpretation, and article drafting. S-IT, TF, AC, YU, and MS critically revised the article for important intellectual content. All the authors contributed to the article and approved the submitted version.

## Conflict of Interest

The authors declare that the research was conducted in the absence of any commercial or financial relationships that could be construed as a potential conflict of interest.
